# Paroxysmal nocturnal hemoglobinuria presenting as cerebral venous sinus thrombosis: a case report

**DOI:** 10.1186/1755-7682-7-39

**Published:** 2014-08-15

**Authors:** Abdul Rauf Memon, Rizwan Khan, Mohammad Uzair Abdul Rauf, Kashif Shafique

**Affiliations:** 1 Department of Medicine, Dow University of Health Sciences, Karachi, Pakistan; 2 School of Public Health, Dow University of Health Sciences, Karachi, Pakistan; 3 Institute of Health and Wellbeing, Public Health, University of Glasgow, 1-Lilybank Gardens, Glasgow G12 8RZ, UK

## Abstract

Paroxysmal Nocturnal Hemoglobinuria (PNH) is a rare type of acquired hemolytic anemia that is frequently associated with thrombophilia. It may rarely present with cerebral venous sinus thrombosis, which manifests clinically with signs of raised intracranial pressure and requires lifelong anticoagulation therapy. One such rare presentation was seen in a 28 years old male who had history of recurrent episodes of passing red colored urine and this time presented with severe headache. He was diagnosed to have cerebral venous sinus thrombosis and on further workup was found to be suffering from PNH.

## Introduction

PNH is a rare acquired clonal hematopoietic stem cell disorder characterized by abnormal sensitivity of red blood cells to lysis by complement. It is caused by genetic mutation resulting in deficiency of glycosyl phosphatidylinositol anchor (GPA) for cell membrane proteins including complement regulating proteins CD55 and CD59 [1]. PNH occurs in all populations throughout the world but it is a rare disease with prevalence estimated to be up to 5 per million [2]. It is considered unique condition in a sense that its manifestations may include hemolytic anemia (due to acquired intracorpuscular defect), pancytopenia (due to marrow failure) and tendency to have venous thrombosis [2]. Hemolysis occurs throughout the day but patients may present for passing red concentrated urine in the morning. As urine is more concentrated in the morning, this is when color is more pronounced. The hypothesis of increased hemolysis at night during sleep due to acidosis or low steroid levels is not supported by studies. The gold standard diagnostic test for PNH is flow cytometry of RBCs to demonstrate absent or reduced expression of both CD55 and CD59 [3].

Patients with PNH experience a high incidence (14-40%) of thrombotic events, mostly venous and rarely arterial [4]. Thrombotic events in PNH may occur despite thrombocytopenia or pancytopenia and they have a predilection for unusaual locations in the venous system. The vessels mostly involved are visceral veins (hepatic, portal, mesenteric, splenic, and renal veins), followed by cerebral and dermal veins. Here we report a case of young male who had history of recurrent episodes of passing red colored urine and this time also presented with severe headache.

## Case report

A 28 years old male, resident of Karachi - Pakistan, worker in a towel factory, came with two years history of recurrent episodes of dark red colored urine, dysphagia for one month and severe frontal headache with blurring of vision and vomiting for one week. On examination he was hemodynamically stable and moderately anemic. Neurological examination revealed Glasgow Coma Scale (GCS) 15/15, normal power, extensor right plantar response and bilateral papilledema on fundoscopic examination. Spleen and liver were not palpable on abdominal examination and rest of the examination was unremarkable.

**Figure 1:**
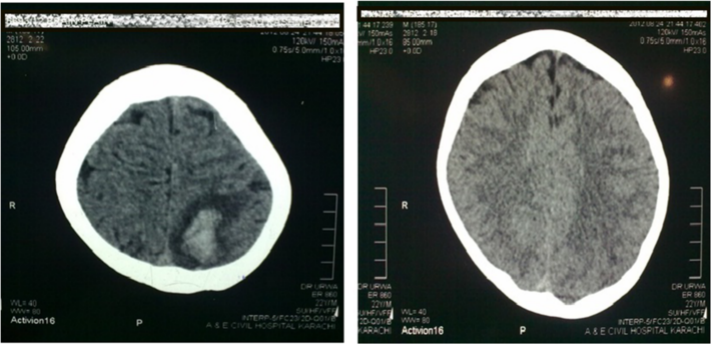
Computed Tomographic (CT) scan of brain showing hemorrhagic infarct.

**Figure 2:**
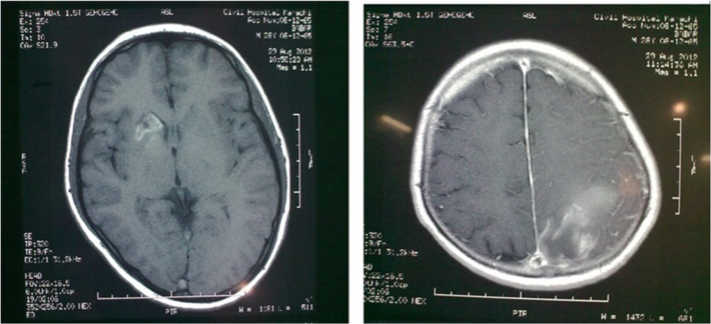
Magnetic Resonance Imaging (MRI) brain revealing multiple hemorrhagic infarcts.

**Figure 3:**
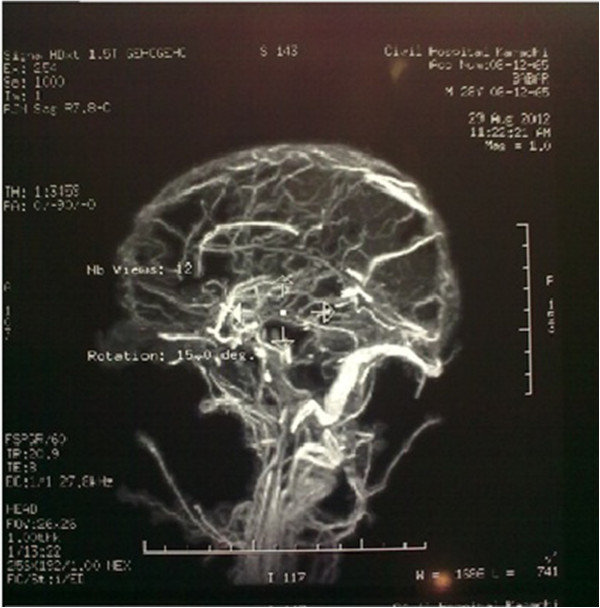
Magnetic Resonance Venography (MRV) brain revealing extensive venous sinus thrombosis.

Laboratory investigations showed hemoglobin of 5.8 g/dl with MCV 96 fl, reticulocyte count 3% (corrected 1%) and normal platelet and total leukocyte count. Serum bilirubin was slightly raised with predominant indirect bilirubin component with normal Alanine Aminotransferase (ALA), alkaline phosphatase, urea, creatinine and electrolyte levels. Lactate Dehydrogenase (LDH) came out to be markedly increased (1467 mg/dl). Urine detailed report showed red cells with no casts. Ultrasound abdomen including kidneys and urinary bladder were normal.Computerized Tomography (CT) of scan brain was done on the day of admission that showed a hyper-intense lesion in left parietal region, intra-cerebral hemorrhage and hemorrhagic infarct (Figure 1). Meanwhile patient developed generalized tonic-colonic seizures. He was managed accordingly with antiepileptic and analgesic drugs. Magnetic Resonance Imaging (MRI) of brain including Magnetic Resonance Venography (MRV) was performed. Blood was collected to perform battery of tests before transfusion. The MRI brain revealed a hematoma in left parietal region with another small hemorrhage in right basal ganglia (Figure 2).On MR venography a filling defect was identified in superior sagittal sinus, confluence of sinuses, straight sinus, transverse and sigmoid sinuses on right side, extending into proximal internal jugular vein. Radiological signs confirmed the diagnosis of extensive right sided dural venous sinus thrombosis (Figure 3). Patient was started with anticoagulation therapy along with 3rd generation cephalosporin and his condition improved gradually. Third generation Cephalosporinwas used to treat infectionas they have broad spectrum coverage and adequate CNS penetration.

The hematological tests sent earlier showed negative Coombs’ test and both Glucose 6 Phosphate Dehydrogenase (G6PD) levels and hemoglobin electrophoresis were normal. The flow cytometry done on RBCs revealed CD55 negative and CD59 double population, confirming the diagnosis of PNH. Patient was started on heparin and warfarin and discharged with warfarin when International Normalized Ratio (INR) was achieved with target range. He was also started with steroids and hematinic supplementation and is currently on outpatient follow up. Eculizumab could not be used because of its non availability.

## Discussion

Although thrombophilia is the leading cause of morbidity and mortality in PNH [5], exact mechanism of this tendency is still unclear. The pathogenesis is probably multifactorial. The major contributor is endothelial damage caused by free hemoglobin and possibly complement system [6]. Release of free hemoglobin activates the endothelium and scavenges Nitric Oxide. In addition complement mediated damage of Glycosyl Phosphatidyl Inositol (GPI) deficient blood cells may result in the release of procoagulant micro particles into the circulation and platelet activation. The risk of venous thrombotic events is said to be directly related to the size of PNH clone. Patients with less than 50% PNH granulocytes seldom develop thrombosis, whereas patients with larger clone sizes appear to be at great risk and will require anticoagulation [7]. Cerebral venous sinus thrombosis (CVST) in PNH usually involves superior sagittal sinus producing edematous, congested cortex and a tendency for hemorrhagic infarction. It clinically presents with signs and symptoms of raised intracranial pressure (headache, vomiting and papilledema). The gold standard diagnostic test for CVST is MR venography.

The treatment options for hemolytic anemia in PNH include blood transfusions, pulse prednisolone for acute attacks, folic acid, iron supplements, low dose prednisolone and eculizumab (humanized monoclonal antibody against complement C5) for chronic hemolysis. Iron replacement can stimulate reticulocytosis that can trigger hemolysis by releasing new cohort of complement sensitive cells. This can be prevented by adding prednisone during replacement therapy. Intravascular hemolysis is the dominant feature of classic PNH, and this process is blocked by the complement inhibitor eculizumab with decreased need of blood transfusions and marked improvement in signs and symptoms and quality of life [8,9]. The thrombotic tendency of PNH also appears to be ameliorated by eculizumab. The 5 year survival of patients with PNH prior to eculizumab therapy was 67% and has improved to 96% in patients who used the monoclonal antibody. The medication also decreased the risk for thrombotic events from 6% per year to less than 1% per year. It has been shown that eculizumab therapy, which is effective in decreasing hemolysis, can also decrease the risk for venous thrombosis [10,11]. Continuation of anticoagulation in patients with PNH with a previous thrombosis while on eculizumab is recommended as stopping therapy has not been studied. The drug has no effect on the bone marrow failure component of the disease.

All PNH patients who experience a thrombotic complication are candidates for indefinite anticoagulation. Concomitant intracranial hemorrhage is not a contraindication for anticoagulation. In a retrospective study on 102 patients with Cerebral Venous Sinus Thrombosis (CVST), 43 had an intracranial hemorrhage. Among 27 (63%) who were treated with dose adjusted intravenous heparin after Intracranial Hemorrhage (ICH), 4 (15%) died and 14 patients (52%) recovered completely. Of the 13 patients who did not receive heparin, mortality was higher (69%) with lower improvement in functional outcome (only 3 patients recovered completely) [12]. Anticoagulation prophylaxis against thromboembolic events without prior thrombotic complication is advocated by many clinicians, but due to lack of randomized, prospective studies this is still an issue of debate. Up till now heterologous bone marrow transplantation is the only curative procedure for PNH. However the transplant related mortality is high, that is 42% [13], so this form of therapy is reserved for those who are severely hypoplastic and refractory to other forms of treatment.

### Consent

Written informed consent was obtained from patient for publication of this case report and any accompanying images.

## Abbreviations

PNH: Paroxysmal Nocturnal Hemoglobinuria (PNH); CVST: Cerebral venous sinus thrombosis.

## Competing interests

The author’s declared no potential competing interest with respect to the research, authorship, and/or publication of this article.

## Authors’ contributions

ARM, MR, MAUR, KS conceived the idea. ARM, MR and MAUR were major contributors to the writing of the manuscript. ARM and MR diagnosed and treated the patient. All authors read and approved the final manuscript.
